# Immunogenicity and Safety of Adjuvanted Recombinant Zoster Vaccine in Rheumatoid Arthritis Patients on Anti-Cellular Biologic Agents or JAK Inhibitors: A Prospective Observational Study

**DOI:** 10.3390/ijms24086967

**Published:** 2023-04-09

**Authors:** Vincenzo Venerito, Pasquale Stefanizzi, Luca Cantarini, Marlea Lavista, Maria Grazia Galeone, Antonio Di Lorenzo, Florenzo Iannone, Silvio Tafuri, Giuseppe Lopalco

**Affiliations:** 1 Department of Precision and Regenerative Medicine and Ionian Area (DiMePRe-J), Polyclinic Hospital, University of Bari, 70124 Bari, Italy; 2 Interdisciplinary Department of Medicine, Aldo Moro University of Bari, 70121 Bari, Italy; 3 Department of Medical Sciences, Surgery and Neurosciences, Research Center of Systemic Autoinflammatory Diseases and Behçet’s Disease Clinic, University of Siena, 53100 Siena, Italy

**Keywords:** rheumatoid arthritis, vaccine, herpes zoster, biological agents, personalized medicine

## Abstract

Rheumatoid arthritis (RA) patients on JAK inhibitors (JAKi) have an increased HZ risk compared to those on biologic DMARDs (bDMARDs). Recently, the Adjuvanted Recombinant Zoster Vaccine (RZV) became available worldwide, showing good effectiveness in patients with inflammatory arthritis. Nevertheless, direct evidence of the immunogenicity of such a vaccine in those on JAKi or anti-cellular bDMARDs is still lacking. This prospective study aimed to assess RZV immunogenicity and safety in RA patients receiving JAKi or anti-cellular bDMARDs that are known to lead to impaired immune response. Patients with classified RA according to ACR/EULAR 2010 criteria on different JAKi or anti-cellular biologics (namely, abatacept and rituximab) followed at the RA clinic of our tertiary center were prospectively observed. Patients received two shots of the RZV. Treatments were not discontinued. At the first and second shots, and one month after the second shot, from all patients with RA, a sample was collected and RZV immunogenicity was assessed and compared between the treatment groups and healthy controls (HCs) receiving RZV for routine vaccination. We also kept track of disease activity at different follow-up times. Fifty-two consecutive RA patients, 44 females (84.61%), with an average age (±SD) of 57.46 ± 11.64 years and mean disease duration of 80.80 ± 73.06 months, underwent complete RZV vaccination between February and June 2022 at our center. At the time of the second shot (1-month follow-up from baseline), anti-VZV IgG titer increased significantly in both groups with similar magnitude (bDMARDs: 2258.76 ± 897.07 mIU/mL; JAKi: 2059.19 ± 876.62 mIU/mL, *p* < 0.001 for both from baseline). At one-month follow-up from the second shot, anti-VZV IgG titers remained stable in the bDMARDs group (2347.46 ± 975.47) and increased significantly in the JAKi group (2582.65 ± 821.59 mIU/mL, *p* = 0.03); still, no difference was observed between groups comparing IgG levels at this follow-up time. No RA flare was recorded. No significant difference was shown among treatment groups and HCs. RZV immunogenicity is not impaired in RA patients on JAKi or anti-cellular bDMARDs. A single shot of RZV can lead to an anti-VZV immune response similar to HCs without discontinuing DMARDs.

## 1. Introduction

Rheumatoid arthritis (RA) patients are at an increased risk of infections due to various factors, including aberrant immune response due to neutrophil margination and constricted T cell receptor repertoire, eventual accompanying comorbidities such as chronic kidney disease and diabetes, uncontrolled disease activity, and immunosuppressive drug usage such as steroids and disease-modifying anti-rheumatic drugs (DMARDs) [1,2]. Robust evidence has shown that the latter factors contribute to making the overall infection risk of RA 2-fold higher than the general population [1]. Opportunistic infection incidence has been reported to be 3-fold higher in RA patients, being approximately 3.67 per 100 PY [3,4,5]. Some DMARDs have been widely used as potent immunosuppressive agents to treat RA, and they contribute to increased infection risk [5]. Corticosteroid use also increases serious infections by 2–4-fold in a dose-dependent manner [5]. Several studies have shown that biologic DMARD use is related to serious bacterial infections [6] requiring hospitalization in the first year of treatment. In one meta-analysis, biologic DMARDs were associated with the development of opportunistic infections in RA patients due to an increased risk of mycobacterial and viral infections including herpes zoster (HZ) [5]. In this regard, one of the most prevalent opportunistic pathogens is Varicella-Zoster Virus (VZV) [3]. HZ IRs for bDMARDs in patients with RA varied from 1.95 to 2.71 per 100 PY, with the highest IR reported for infliximab, and risk relative to abatacept similar across all bDMARDs [7]. In immunocompromised individuals, VZV can reactivate more efficiently, leading to a higher risk of HZ and more severe symptoms. VZV reactivation machinery employs counter mechanisms to prevent the induction of anti-viral interferon-stimulated genes by inhibiting interferon-stimulated JAK-STAT signaling [8]. Of note, RA patients on JAK inhibitors (JAKi) also have an increased HZ risk than those on biologic DMARDs (bDMARDs), possibly due to impaired interferon I signal transduction [9]. For many years, anti-VZV vaccination strategy had been challenging due to contraindications to administering the anti-VZV live-attenuated vaccine in patients on bDMARDs or JAKi [10]. Pooled analyses of data from baricitinib RA clinical studies reported an overall HZ IR of 3.0 per 100 PY for all baricitinib [7] and HZ IRs of 4.3 per 100 PY for baricitinib 4 mg and 1.0 per 100 PY for placebo over the placebo-controlled period (26), and an overall HZ IR of 6.5 per 100 PY for Japanese patients [7]. Similarly, for tofacitinib, an increased risk of HZ was reported for patients from Asia, with HZ IRs of 8.0 and 8.4 per 100 PY for patients with RA from Japan and Korea, respectively, vs. 4.0 per 100 PY across the tofacitinib RA clinical program [7]. Recently, the Adjuvanted Recombinant Zoster Vaccine (RZV) became available worldwide, showing good effectiveness in the ZOE trial programs in patients affected with inflammatory arthritis, including RA and spondyloarthritis [11]. Nevertheless, to date, direct evidence of the immunogenicity of such a vaccine in those on JAKi or anti-cellular bDMARDs is still lacking. This is of utmost importance for adapting and personalizing RA patient management and vaccination strategy.

This prospective study aimed to assess RZV immunogenicity and safety in RA patients receiving JAKi or anti-cellular bDMARDs that are possibly known to lead to impaired immune response.

## 2. Results

**Figure 1:**
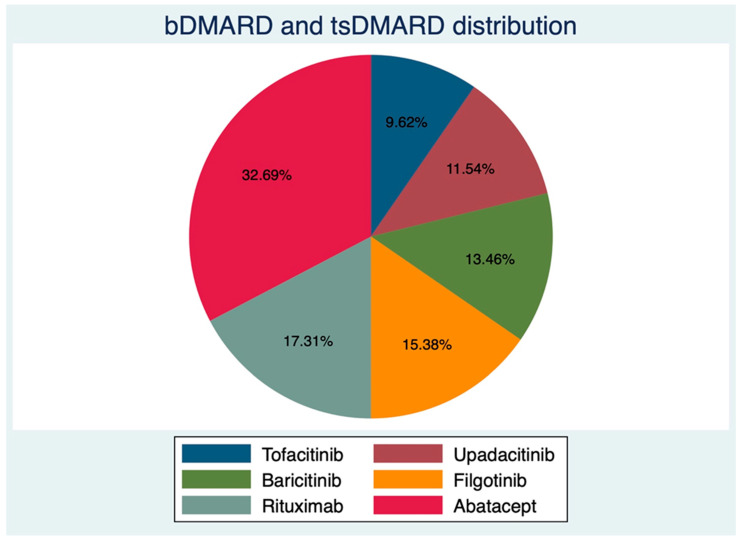
Distribution of biological and targeted synthetic disease-modifying anti-rheumatic drugs.

Of 52 consecutive RA patients, 44 females (84.61%), with an average age (±SD) of 57.46 ± 11.64 years and mean disease duration of 80.80 ± 73.06 months, underwent complete RZV vaccination between February and June 2022 at our center. There were 26 patients on JAKi at RZV baseline; in particular, 8 out of 52 (15.38%) were on Filgotinib (FIL), 7/52 (13.46%) on Baricitinib (BAR), 6/52 (11.54%) on Upadacitinib (UPA), and 5/52 (9.62%) on Tofacitinib (TOF). Those on anti-cellular bDMARDs were mostly on ABA (17/52, 32.69%) and RTX (9/52, 17.31%). The distribution of bDMARDs and targeted synthetic (ts-) DMARDs is shown in Figure 1. Even though the two groups were not different according to gender distribution, age, and BMI (Table 1), those on bDMARDs had longer disease duration (97.88 ± 82.48 vs. 63.85 ± 59.03 months, *p* = 0.04), lower HAQ-DI (median (IQR) 0.87 (0.25–1.87) vs. 0.5 (0.12–1.12)), and a trend towards higher disease activity (3.32 ± 1.39 vs. 2.88 ± 1.01, *p* = 0.06). Patients in the bDMARDs group also had a higher prednisone equivalent dose (PDNeq) (3.75 ± 3.70 vs. 2.28 ±2.25, *p* =0.04). Complete patient characteristics are shown in Table 1.

**Figure 2:**
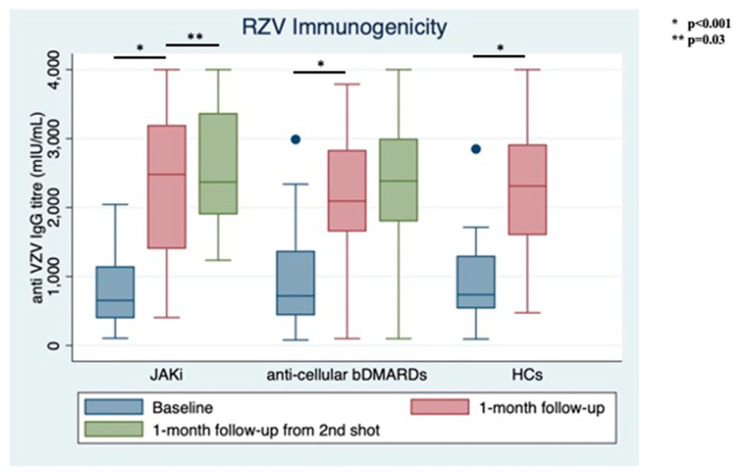
Immunogenicity of recombinant Adjuvanted Recombinant Zoster Vaccine (RZV) at different follow-up times stratified between treatment groups and compared with healthy controls (HCs).

Only 2 patients (3.85%) reported previous HZ, with both in the bDMARDs group. No patients had ever received the live zoster vaccine. At RZV baseline, the anti-VZV IgG serum level was similar between groups (1000.66 ± 140.684 mIU/mL for those on bDMARDs vs. 843.96 ± 114.70 mIU/mL JAKi patients, *p* = 0.39, Figure 2). RZV reactogenicity at the first shot is shown in Table 1.

Of the JAKi group, 21/26 (RR = 80.77 × 100 follow-up) and 24/26 (RR = 92.31 × 100 follow-up) of those receiving bDMARDs complained of local site reactions with either redness, swelling, and/or skin thickening. Fatigue (13/52, RR = 25 × 100 follow-up), fever (11/52, 21.15 × 100 follow-up), and headache (11/52, 21.15 × 100 follow-up) were the most prevalent short-term reactions.

At the time of the second shot (1-month follow-up from baseline), anti-VZV IgG titer increased significantly in both groups with similar magnitude (bDMARDs: 2258.76 ± 897.07 mIU/mL; JAKi: 2059.19 ± 876.62 mIU/mL, *p* < 0.001 for both from baseline). Only 3 out of 52 (5.77%) patients did not show vaccine response: 1 in the JAKi group (3.85%) and 2 in the bDMARDs group (7.69%).

At one-month follow-up from the second shot, anti-VZV IgG titers remained stable in the bDMARDs group (2347.46 ± 975.47) and increased significantly in the JAKi group (2582.65 ± 821.59 mIU/mL, *p* = 0.03); still, no difference was observed between groups comparing IgG levels at this follow-up time (*p* = 0.12, Figure 2).

Of note, the individual not achieving response with the first jab in the JAKi group displayed a good response with the second shot; conversely, none of the patients who did not develop an anti-VZV immune response succeeded in responding to the RZV booster.

No RA flare was recorded either in the JAKi or the bDMARDs group, with DAS28-ESR remaining stable at each follow-up visit without significant difference. Similarly, HAQ-DI remained stable in both groups (Table 1). No serious adverse events were recorded in the JAKi or the bDMARDs group during the observation period. None of the baseline covariates were associated with anti-VZV IgG level nor with DAS28-ESR at different follow-up visits; interestingly, higher PDNeq was not associated with lower immunogenicity (*p* = 0.38).

During the observation periods, 33 HCs, including 15 females (45.45%) with a mean age (±SD) of 71.22 ± 3.32 years, received RZV at the same vaccine hub. At RZV baseline, HCs’ IgG titer was 982.04 ± 140.12 mIU/mL and significantly increased to 2258.75 ± 155.98 1 month after the first shot. Only one HC did not achieve vaccine response at the first jab (1/33, 3.03%).

No significant difference was shown among treatment groups and HCs, neither at baseline (*p* for Bartlett’s test = 0.64) nor at the next follow-up time (*p* for Bartlett’s test = 0.49).

## 3. Discussion

While the biologic treatment agents are associated with HZ flare, robust evidence confirmed that RA patients on JAKi have an increased risk of HZ up to 2-fold higher than those on bDMARDs [3]. Given that, the 2019 update of the EULAR recommendation for vaccination in adult patients with inflammatory rheumatic diseases advocated administering the live-attenuated vaccine four weeks before initiating bDMARDs or JAKi, but not during treatment [10]. A new non-live RZV vaccine was licensed in Europe in March 2018 but made available in most countries only recently.

From a pooled post hoc analysis on two parallel randomized trials, namely, ZOE-50/70, RZV appeared highly effective in patients with pre-existing rheumatic diseases such as RA or spondyloarthritis, with a similar frequency of serious adverse events between treatment arms and placebo groups [11]. Being non-live, it had been thought to replace the live-attenuated vaccine in rheumatic patients, following EULAR 2019 recommendations. Indeed, the American College of Rheumatology (ACR) 2022 recommendation strongly recommends RZV for rheumatic patients aged >18 years who are on immunosuppressive medication [12].

Nevertheless, the immunogenicity of such a vaccine has not been explicitly and prospectively tested in RA patients on JAKi or bDMARDs.

We showed that RZV was highly immunogenic with a significant immune response detected at 1 month from the first jab in more than 90% of our cohort. At that follow-up time, only 3 out of 52 patients (5.76%) failed to achieve good response; for one of them, the second jab resulted in good immunogenicity, making a complete RZV vaccination immunogenic in more than 95% of our cohort. Interestingly, the anti-VZV IgG levels increased comparably in patients with JAKi and bDMARDs without a significant difference when assessed at 1 month from baseline and 1 month from the second shot. The magnitude of the elicited immune response was similar to the one obtained in HCs with a single shot.

Of note, we did not discontinue any DMARDs at the time of vaccination; this is particularly important for rheumatologists to adequately schedule vaccination for RA patients on JAKi or anti-cellular biologic agents. Moreover, our results appear surprising yet promising, given what the rheumatology community observed during the pandemic era with the severe impairment of anti-SARS-CoV 2 mRNA vaccines caused by several immunosuppressive agents such as RTX [13,14].

Patients reported RZV to be highly reactogenic, with injection site reactions such as redness, swelling, and/or skin thickening in the vast majority of the cohort. Additionally, up to a third of our cohorts reported fatigue, headache, and fever. In this regard, our results depict a more frequent reactogenicity than previous reports in the literature. Stevens et al. [15], in an extensive retrospective study of 403 patients (239 patients with RA and 164 patients with other rheumatic diseases), observed reactogenic side effects in 51 (12.7%) patients: 43 (10.7%) patients after the first dose and 12 (5.4%) patients after the second one. Such a discrepancy may be due to the prospective fashion of our study where patients were specifically asked for reactogenicity. Nevertheless, no serious adverse events occurred, consistent with previous evidence.

We did not observe an RZV-caused RA flare, with DAS28 remaining stable at different tight follow-up visits, together with HAQ-DI. Consistently, the rate of patients in remission changed neither in the JAKi nor the bDMARDs group during the observation period.

Conversely, Stevens et al. retrospectively recognized a flare in 27/403 rheumatic patients (6.7%, an incidence rate of 6.7 cases per 100 PY), with 23 (5.7%) flares occurring after the first dose and 5 (2.3%) flares occurring after the second. One patient experienced a flare following both doses. There were 19 (5.0%) flares identified in patients with RA. Moreover, Lenfant et al. [16] described a flare of 21/88 RA patients (24%) receiving RZV.

While the last two reports contrast with our data, it is conceivable that some flaws in recording RA flares exist in such retrospective studies, as the flare definition was based on findings in health records, steroid prescriptions, or was patient-reported. Interestingly, no data had been reported on how caring rheumatologists managed conventional synthetic (cs-) DMARDs, biologic agents, and JAKi at the time of vaccination, so drug discontinuation as a cause of a flare cannot be excluded [15,16].

Additionally, in both the American retrospective cohorts, RZV was given as a two-dose series, with doses administered two to six months apart. On the contrary, in the study of Steven et al. [15], RA patients received both doses with a median interval of 2.9 months in between doses according to Lenfant et al. [16]. Our follow-up was tighter and included systematic assessment of disease activity with DAS28. On this basis, at least for flares occurring somewhere between the two shots, it should be acknowledged that the causal relationship between RZV and flares might not appear so robust with such a long interval between the two jabs. In contrast, our follow-up was tighter and included the prospective and systematic assessment of DAS28 one month apart from each shot. Additionally, our study protocol did not require JAKi or bDMARDs to be discontinued.

Our study has several strengths. Our results help to define the safety of RZV immunogenicity in RA patients on JAKi or anticellular bDMARDs. To our best knowledge, this is the first prospective study specifically powered to detect differences in terms of vaccine immunogenicity between different treatment groups and HCs. However, we should acknowledge a large effect size in our a priori power analysis and the lack of observation period for detecting post-vaccine HZ outbreak, even if it was not among the aims of our study. We did not include patients on anti-tumor necrosis factor inhibitors (TNFi), as evidence suggests that such a class is unlikely to impair vaccine immunogenicity significantly [17,18]. Additionally, although RZV should lead to a glycoprotein-E (gE) specific response, we used an assay developed on a partially purified extract of infected cell cultures (ROD strain), hence containing a great variety of antigens, including glycoprotein-E. However, we acknowledge that our immunogenicity assessment method differed from the one used in ZOE-50 and ZOE-70 trials, where specifically anti-gE antibody concentrations were measured by anti-gE enzyme-linked immunosorbent assay [19].

Additionally, we acknowledge the lack of evaluation of the CD4+ T-cell immune response playing a central role in preventing VZV reactivation [11].

In conclusion, we showed that RZV immunogenicity is not impaired in RA patients on JAKi or anti-cellular bDMARDs. A single shot of RZV can lead to an anti-VZV immune response similar to HCs without the need to discontinue DMARDs.

RZV reactogenicity is frequent but mild, and patients should be advised of that; nevertheless, we detect no RA flare on a tight follow-up schedule. This study provides a helpful insight for refining the anti-VZV vaccination strategy in RA patients on JAKi or non-TNFi bDMARDs.

## 4. Material and Methods

### 4.1. Data Gathering

Patients with classified RA according to ACR/EULAR 2010 criteria on JAKi or anti-cellular biologics (namely, abatacept (ABA) and rituximab (RTX)) followed at the RA clinic of our tertiary center were prospectively observed from February to June 2022.

Our institution set up a vaccination campaign during which patients with RA received two shots of the RZV (Shingrix^®^, GlaxoSmithKline) one month apart. Our study included all consecutive patients who underwent vaccination during such a campaign, aged 18–85 years with active treatment, who gave written informed consent. At the first and second shots, and one month after the second shot, from all patients with RA, a 5 mL serum sample was collected. It was tested by chemiluminescence using LIAISON^®^ VZV IgG, a semi-quantitative method performed with a standardized commercial kit (Diasorin). An anti-varicella IgG titer > 150 mIU/mL was defined as positive (DiaSorin. The Diagnostic Specialist. LIAISON^®^ VZV IgG. The fully automated solution for antibody detection. Available on: https://www.diasorin.com/sites/default/files/allegati_prodotti/ese_scheda_vzv_rev_02_low.pdf accessed on 1 December 2020). Blood samples were collected and analyzed at the same institution’s laboratory.

JAKi and biologic treatment, together with methotrexate (MTX), were not discontinued throughout the whole period. Vaccine response was defined as a 20% increase in anti-VZV IgG level compared to baseline determination. Demographic and clinical characteristics include disease phenotypes, clinimetrics (Disease Activity Score on 28 Joints-Erythrocyte Sedimentation Rate, DAS28-ESR), and patient-reported outcome measures (Health Assessment Questionnaire-Disability Index, HAQ-DI) were recorded at baseline consultation before vaccination from hospital e-health records. At the same time, patients were asked for prior HZ and/or related symptoms. The assessment of DAS28-ESR was also repeated at the time of the second shot and one month after that on hospital e-health records in two consultations at the rheumatology department. At each subsequent visit, patients were asked for the occurrence of swollen joints in the time interval between the consultation on the first and at the second jab, and between the second jab and the 1-month follow-up visit.

Patients aged 65 years and older without autoimmune inflammatory rheumatic diseases, diabetes, cardiovascular diseases, or active immunosuppression who received RZV at the same vaccination hub according to local policy in the abovementioned observation period were considered healthy controls (HCs). Their sera were gathered at the time of the first shot and one month after; all gave their written informed consent. The local ethics committee approved this prospective study as part of the BioPure study (IRB approval n.5277).

### 4.2. Safety

Vaccinated patients were followed up for 7 days in order to detect any adverse events following immunization (AEFIs); for each dose, a post-vaccination diary was implemented in which any adverse reaction was notified [20].

As provided by Italian law, all adverse events were entered into the Italian Pharmacovigilance Network (RNF) database.

In case of access to the structures of the National Health Service, the health documentation was acquired (hospital discharge card, laboratory and/or instrumental test reports, specialist consultations, etc.). The data were stored according to the privacy law.

WHO guidelines have been used to classify AEFIs as ‘serious’ or ‘not serious’. They are considered serious in the case of death, life threat, in-patient hospitalization, prolongation of existing hospitalization, persistent or significant disability/incapacity, congenital anomaly/birth defect, or intervention to prevent permanent impairment or damage. Furthermore, a list of health conditions that must be considered as serious AEFIs was published by the European Medical Agency [21].

For serious AEFIs, the WHO causality assessment algorithm was applied to classify the AEFI as ‘consistent causal association’, ‘inconsistent causal association’, ‘indeterminate’, or ‘not classifiable’. The causality assessment was carried out separately by two public health physicians who were experts in vaccinology; in case of disagreement, a third physician was consulted [22,23].

The adverse events reported were grouped into the following categories:

- Local reactions (pain, redness, swelling, induration at the injection site);
- Lymphadenopathy;
- General malaise (myalgia, fatigue);
- Neurological symptoms (headache);
- Fever/hyperpyrexia.

To calculate the reporting rate (RR), the number of reports was used as the numerator and the number of subjects recruited in this study was used as the denominator; the proportion was subsequently multiplied by 100 and the confidence interval at 95% (95%CI) was reported.

### 4.3. A Priori Power Analysis

For this prospective observational study, we needed 26 RA patients per group (JAKi vs. anti-cellular biologics and RA patients as a whole vs. healthy controls) to test the difference in ant-VZV IgG at α = 0.05, 1 − β = 0.80, and with an effect size of 0.80 (large).

### 4.4. Statistics

We assessed the difference in anti-VZV IgG levels between groups with the Student’s t-test. The paired t-test and McNemar’s test were used to determine the difference between mean DAS28-ESR, HAQ-DI, and remission rate at different time points, respectively. The associations of recorded covariates with IgG levels and DAS28-ESR scores after vaccination were investigated using linear regression. ANOVA with Bartlett’s test was used to assess the difference between serum IgG titers between treatment groups and HCs, with Stata 17 (StataCorp, College Station, TX, USA) used for the analysis.

## Figures and Tables

**Table 1:** Patient characteristics at different follow-up times. * *p* < 0.001 (previous time point, same group), ** *p* = 0.03 (previous time point, same group), ° *p* = 0.04 (group comparison).

	JAKi									Anti-Cellular bDMARDs								
	Baseline			At 2nd Shot			3-Month Follow-Up			Baseline			At 2nd Shot			3-Month Follow-Up		
	Av.obs			Av.obs.			Av.obs.			Av.obs.			Av. Obs.			Av. Obs.		
Female, n%	26	23	88.46							26	21	80.76	26					
Age, years, mean (SD)	26	55.61	13.26							26	59.30	9.67	26					
BMI, mean (SD)	26	26.89	4.44							26	25.05	4.82	26					
DAS28, mean (SD)	26	2.88	1.01	26	2.81	0.99	26	2.61	1.08	26	3.45	1.59	26	3.32	1.39	26	3.09	1.17
Disease duration, months, mean (SD)	26	63.85 °	59.03							26	97.88 °	82.48	26					
HAQ-DI, median (IQR)	26	0.5	0.12–1.12	25	0.5	0.12–1.12	36	0.43	0.12–1.12	26	0.87	0.37–1.87	26	0.87	0.25–1.87	26	0.87	0.25–1.87
RF positivity, n%	26	19	73.08							26	14	53.85	26					
ACPA positivity, n%	26	22	84.62							26	18	69.23	26					
Treatment line, median (IQR)	26	2	1.0–3.0							26	2	1.0–3.0	26					
PDNeq, mg, mean(SD)	26	2.28 °	2.25							26	3.75 °	3.70	26					
MTX combotherapy, n%	26	13	50							26	9	34.62	26					
Previously reported HZ, n%	26	0	0							26	2	7.69	26					
Anti-VZV IgG, miU/mL, mean (SD)	26	843.96	584.87	26	2059.19 *	876.62	26	2582.65 **	821.59	26	1000.66	717.35	26	2258.76 *	897.07	26	2347.46	975.47
DAS28 remission, n%	26	10	38.46	26	11	42.30	26	14	53.84	26	9	34.61	26	9	34.61	26	9	34.61
Fever, n%				26	6	23.08							26	5	19.23			
Myalgia, n%				26	4	15.38							26	1	3.85			
Headache, n%				26	4	15.38							26	7	29.92			
Lymphadenopathy, n%				26	1	3.85							26	0	0			
Sleep disorder, n%				26	3	11.54							26	2	7.69			
Injection site reactions (redness, swelling, skin thickening), n%				26	21	80.77							26	24	92.31			
Fatigue, n%				26	8	30.77							26	5	19.23			
Non-responders, n%				26	1	3.85	26	0	0				26	2	7.69	26	2	7.69

*Abbreviations*: ACPA: Anti-Citrullinated Protein Antibody; BMI: Body Mass Index; CDAI: Clinical Disease Activity Index; csDMARDs: Conventional Synthetic Disease-Modifying Anti-Rheumatic Drugs; DAS28-ESR: Disease Activity Score on 28 joints with Erythrocyte Sedimentation Rate; HAQ-DI: Health Assessment Questionnaire-Disability Index MTX: Methotrexate; PDN: Prednisone; RF: Rheumatoid Factor; VZV: Varicella Zoster Virus.

## Data Availability

Data are available upon request to corresponding author.
